# The Diagnostic Significance of Calcium Elimination

**Published:** 1914-06

**Authors:** 


**The Diagnostic Significance of Calcium Elimination.** Rodillon claims (*La Semaine Médicale,* September 10, 1913) that excessive elimination of calcium occurs in some diseases, notably tuberculosis, and the detection of this form of demineralization may be an early guide to the diagnosis of the infection before definite signs have developed.

The alkaline earthy bases of calcium and magnesium are eliminated chiefly by the intestines and the kidneys, and in negligible amounts by the skin, lungs, uterus and in the tears, saliva and semen.

To estimate the amount of calcium eliminated without estimating the amount ingested will of course give erroneous results. Vegetables are rich in calcium, whereas milk contains a relatively small amount and meat very little. Another important point to consider is that the vegetable salts are eliminated chiefly through the intestines while the calcium ingested with meat is eliminated through the kidneys. The explanation is found in the solubility of the substances of combustion. Meat netabolism gives rise to acids eliminated in the form of soluble acid phosphates; the vegetable proteids form alkaline bases and phosphoric acids which unite to form insoluble salts, bicalcic and tricalcic phosphates, which are not dialyzed and therefore expelled in the feces.

To obtain a fair estimate of the calcium eliminated it would be necessary to make a quantitative analysis of both the feces and urine. The former is troublesome and often impracticable, whereas the latter can be tested at the bedside of the patient Rodillon suggests an exclusive meat diet before the test and gives a simple method for estimating the calcium in the urine.

The apparatus for the "calcium reaction" consists of a graduated, flat-bottomed glass cylinder, 15 millimeters in diameter; a dropper of 20 drops (of water) to the cubic centimeter; a white card on which has been traced in black ink a straight line 3 millimeters in cross-section. The reagent, oxalate solution, is made up of: Neutral ammonium oxalate, 3 gm., glacial acetic acid, 5 gm., and distilled water, 40 gm. In the actual test no other reagent is used, but to calibrate the cylinder, or calcimeter," a second solution is employed which is made up of: Calcium carbonate, 0.357 gm.; acetic acid, 1 gm., and distilled water to 1000 gm. Five c.c. each of the reagent and the calcium carbonate solution are poured into the cylinder, mixed thoroughly and allowed to stand for five minutes. The cylinder is then placed vertically over the black line on the card, and enough of the contents removed with the dropper to make the black line visible through the calcium oxalate precipitate. The amount is then read off on the tube and divided by 5 to get the constant. Thus:
read off on the tube and divided by 5 to get the constant.

In testing the urine the calcium carbonate solution is replaced by the urine; the figure read off, say 3.2 c.c., is divided into the constant and the resultant constitutes the amount of calcium oxide in 1 liter of urine.

The normal amount for an adult on a mixed diet is from 0.35 to 0.5 gm. daily. If the figure rises to 0·7 on a mixed diet, 0.9 gm. on a meat diet, or 0.5 gm. on a vegetable diet, it indicates demineralization; and the condition is suspicious of tuberculosis.

